# Pentraxin-3 and Lipocalin-2 are decreased in patients with uveal effusion syndrome

**DOI:** 10.1007/s10792-025-03835-5

**Published:** 2025-11-05

**Authors:** Caroline Havertz, Benedikt Schworm, Jakob Siedlecki, Wolfgang Wilfert, Bernd Northoff, Siegfried G. Priglinger, Leonie F. Keidel

**Affiliations:** 1 Department of Ophthalmology, LMU University Hospital, Ludwig Maximilians-University Munich, Mathildenstrasse 8, 80336 Munich, Germany; 2 Institute of Laboratory Medicine, LMU University Hospital, LMU Munich, Munich, Germany

**Keywords:** Choroid, Pachychoroid, Uveal effusions syndrome, Pentraxin-3, Lipocalin-2, Scleral thickness

## Abstract

**Purpose:**

Uveal effusion syndrome (UES) is characterized by exudative detachments of choroid, ciliary body and retina. Like central serous chorioretinopathy (CSCR) it is associated with scleral thickening inhibiting fluid outflow. Recently, decreased levels of Lipocalin-2 (LCN2) and Pentraxin-3 (PTX3) were found in CSCR. The aim of this study was to measure these parameters in patients with UES to evaluate a possible pachychoroid continuum with UES as the maximum form.

**Design:**

A randomized prospective case–control study was performed at the Ludwig Maximilians University, Department of Ophthalmology.

**Methods:**

In patients with UES and in an age- BMI- and sex-matched control group, subfoveal choroidal thickness (SFCT) on optical coherence tomography imaging and the serum levels of PTX3 and LCN2 were measured.

**Results:**

12 patients were included in each group. SFCT was significantly thicker in the UES group compared to the normal controls (*p* = 0.0003). LCN2 and PTX3 values tended to be lower in patients with UES (LCN2: 65.74 ± 33.81 (33.7 − 165.4) ng/ml vs. 108.8 ± 67.1 (40.8 − 261.9, *p* = 0.133) ng/ml PTX3: 0.82 ± 0.52 (0.13 − 2.1) ng/ml vs. 1.23 ± 0.77 (0.3 − 1.86, *p* = 0.260) ng/ml.

**Conclusion:**

Lipocalin-2 and Pentraxin-3 values, which are known to be induced by glucocorticoids, tend to be lower in patients with UES. The results suggest an impairment in the glucocorticoid receptor pathway and underline the hypothesis of a common pathophysiological pathway of CSCR and UES.

## Introduction

Uveal effusion syndrome (UES) is a rare ocular disease, which is defined as spontaneous, non-inflammatory exudative choroidal detachment with or without serous retinal detachment, attributed to impaired transscleral fluid egress and/or vortex vein congestion [1, 2]. A thickened glycosaminoglycan-rich sclera is typical for patients with UES, which is suggested to be the cause for fluid retention in the choroidal space [3]. Another hypothesis is, that the rigid thickened sclera causes a compression of the vortex veins, which in turn leads to backflow into the choroid and remodeling of the veins [4]. This hypothesis is supported by early work of Brockhurst et al., who described decompression of the vortex veins as a successful therapeutic option for UES [5]. Since scleral thickness increases with decreasing axial length, an association of UES with short axial length has previously been reported [6].UES occurs predominantly in men [7]. The exact cause of this gender bias remains unclear.

Central serous chorioretinopathy (CSCR) represents the second stage of the pachychoroid disease spectrum [8]. A thickening of the choroidal Haller layer, the resulting increased hydrostatic pressure, a thinning of the overlying Sattler layer of the choroid and the resulting ischemic environment lead to changes in the RPE (retinal pigment epithelium, pachychoroid pigment epitheliopathy stage). If focal defects develop between the tight junctions within the RPE, fluid can ultimately flow into the subretinal space and diffuse leakage occurs. Recent studies, including one from our group, suggest that a thickened sclera may represent an ocular outflow resistance for the vortex veins in eyes with CSCR and secondarily cause a chronic overload of the venous system, congruent with the pathophysiology of UES [3, 9].

That is to say that both diseases are based on similar morphological and pathophysiological findings and a pachychoroid disease continuum is currently assumed, whereby UES could represent the maximum form [8]. Like CSCR, UES typically affects middle-aged men, is associated with hypermetropia and presents with diffuse exudations in the subretinal space. In both diseases a significantly thickened choroid with specific pachychoroid features (such as thinning of the Sattler layer and thickened Haller veins) and a probably causative thickened sclera have been reported [1, 9].

The question arises whether, in addition to this structural, ocular changes, systemic factors contribute to the etiology of the diseases. Bousquet and colleagues recently observed significantly increased CRP levels and significantly reduced levels of pentraxin-3 (PTX3) and lipocalin-2 (LCN2) in patients with CSCR [10, 11].

PTX3, currently emerging as a novel player in neurodegenerative disorders of the retina is an acute-phase protein that modulates inflammatory processes, [12] similar to LCN2, which is involved in the innate immune response. Previous studies observed a protective effect of both LCN2 and PTX3 against oxidative stress on RPE-cells [13–15]. Transcription of both proteins is essentially controlled by dexamethasone and thus the levels of LCN2 and PTX3 correlate with the activation of the glucocorticoid signaling pathway[16].

The aim of this study is to compare the serum levels of LCN2 and PTX3 in patients with UES to a healthy control cohort. If as in CSCR, lower values are also found in patients with UES, the hypothesis of a similar pathophysiology could be supported further. The parameter could be used in follow-up studies to establish a possible systemic biomarker for evaluating disease activity.

## Material and methods

For this prospective case–control study, 12 patients (12 eyes) with UES at the Ludwig Maximilians-University Munich, Department of Ophthalmology, Germany, were recruited and included into this study from May 2024 to September 2024. Epidemiological and clinical data was obtained from each patient, including age, gender, BMI, previous ocular comorbidities or procedures and objective refraction-based Snellen chart best corrected visual acuity (BVCA), which was later converted to logarithm of the minimum angle of resolution (logMAR) for analysis.

**Fig. 1:**
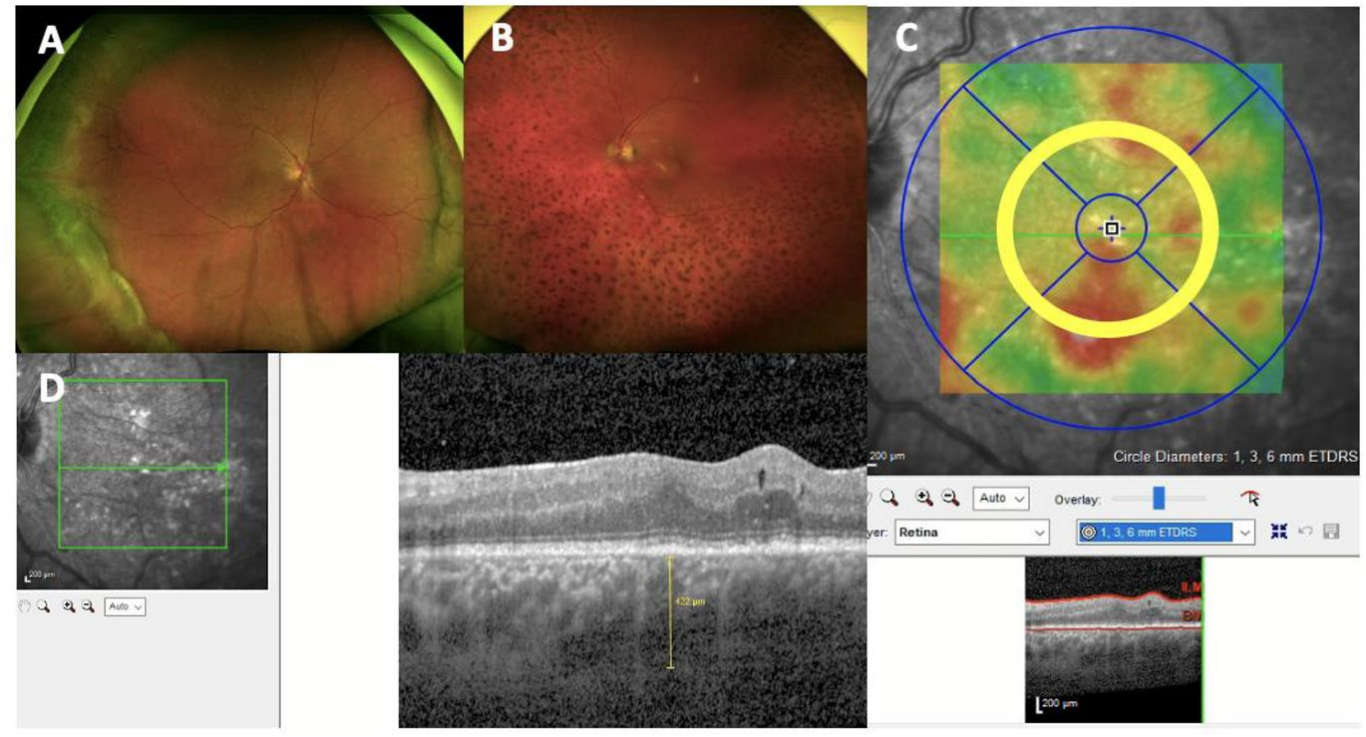
Representative Optos retinal imaging (Optos GmbH, Dunfermline, UK) and macu-lar SD-OCT scans of three patients with UES. **A** Classic picture of a peripheral bullous chorioretinal detachment, **B** Degenerative leopard spots in a chronic pa-tient after chorioretinal detachment, **C** 3 mm area for calculation of CRV and CRT, **D** Measurement of the SFCT (yellow line) in the same patient

Only adult patients with the classic picture of UES in funduscopy, optical coherence tomography (OCT) and autofluorescence were included (Fig. 1A). For diagnosis, the diagnostic criteria of Uyama and colleagues had to be present [2]: A bullous retinal detachment, without any retinal break; shifting of the subretinal fluid with change in head position; absence of leakage between the choroid and the subretinal space on angiography; retinal detachment with additional annular peripheral ciliochoroidal detachment; absence of scleral depression in the area of the ora serrata; exclusion of other reasons for the retinal detachment, such as hypotony, intraocular tumor, rhegmatogenous retinal detachment, and intraocular inflammation.

This group was then matched by age, BMI- and sex to a randomly recruited normal control group (12 patients, 12 eyes) without any retinal disease from the same time span. Patients with a BMI > 35 kg/m^2^ or liver, kidney or severe cardiovascular diseases were excluded from analysis as these pathologies can distort PTX3 and LCN2 values. Also, patients suffering from chronic inflammatory and infectious diseases and patients with an active tumor disease, were excluded from analysis.

### Blood sample

For the evaluation of PTX3, blood plasma was obtained by using EDTA as an anticoagulant. For the evaluation of LCN2, heparin was used as an anticoagulant. According to the manufacturer’s instructions, 20 µl undiluted EDTA plasma was used for PTX3 and 1:20 dilution of heparin plasma (12.5 µl plasma + 237.5 µl RD5-24 Calibrator Diluent) was used for LCN2. The EDTA and heparin blood samples were centrifuged for 15 min between 2 and 8° after collection and frozen at − 20 °C until analysis. ELISA quantikine tests from R&D systems were used for the subsequent analysis (Human Pentraxin 3/TSG-14 ELISA Kit—Quantikine, catalog: DPTX30B and Quantikine Immunoassay Control Set1076 for Human Lipocalin-2, catalog: QC115). Three measurements were performed for each sample and the mean value was determined. To exclude confounding factors, liver, and kidney function parameters as well as the CRP values of the subjects were determined. The tests were carried out at the Central Laboratory at the Ludwig Maximilian University, Campus Großhadern, Institute of Laboratory Medicine. The serum values of LCN2 and PTX3 were pseudonymously compiled with demographic patient data (age, gender, age at first diagnosis). The evaluation data was forwarded directly to the physicians taking part in the study without intermediate transmission to third parties.

### Imaging

Imaging included an acquisition of a standard macular volume scan consisting of 49 equally spaced B-scans covering 20 × 20 degrees centered on the fovea (20° × 20° [5.9 × 5.9 mm], 49 horizontal B-scans, resolution: 512 pixels [X] × 496 pixels [Z], 18 ≤ ART ≤ 30, on Spectralis HRA + OCT, Heidelberg Engineering, Heidelberg, Germany). Subfoveal choroidal thickness (SFCT) was assessed by manual measurements of the distance between Bruch’s membrane and the choroidal–scleral interface below the foveola (Fig. 1D). The central retinal volume (CRV) and central retinal thickness thickness (CRT) were calculated in a 3 mm diameter cylinder centered to the fovea derived from the same macular volume scan (Fig. 1C).

### Statistical analysis

All data were recorded and analyzed in Microsoft Excel spreadsheets (version 16.66.1 for Mac; Microsoft, Redmond, WA, USA). Statistical analysis were performed with Graph Pad Prism (version 10.4.0., LLC, RRID:SCR_002798) and R (version 4.2.0, RRID:SCR_001905). The level of statistical significance was defined as *p* < 0.05. To test for a normal distribution of the two groups, the Kolmogorov–Smirnov test was used. Statistical analyses of the differences between the two groups were performed using the Wilcoxon-Mann–Whitney test. We calculated Pearson correlation coefficients to assess the relationships between CRT, CRV, SFCT, and BCVA and the serum levels of PTX3 and LCN2.

## Results

**Fig. 2:**
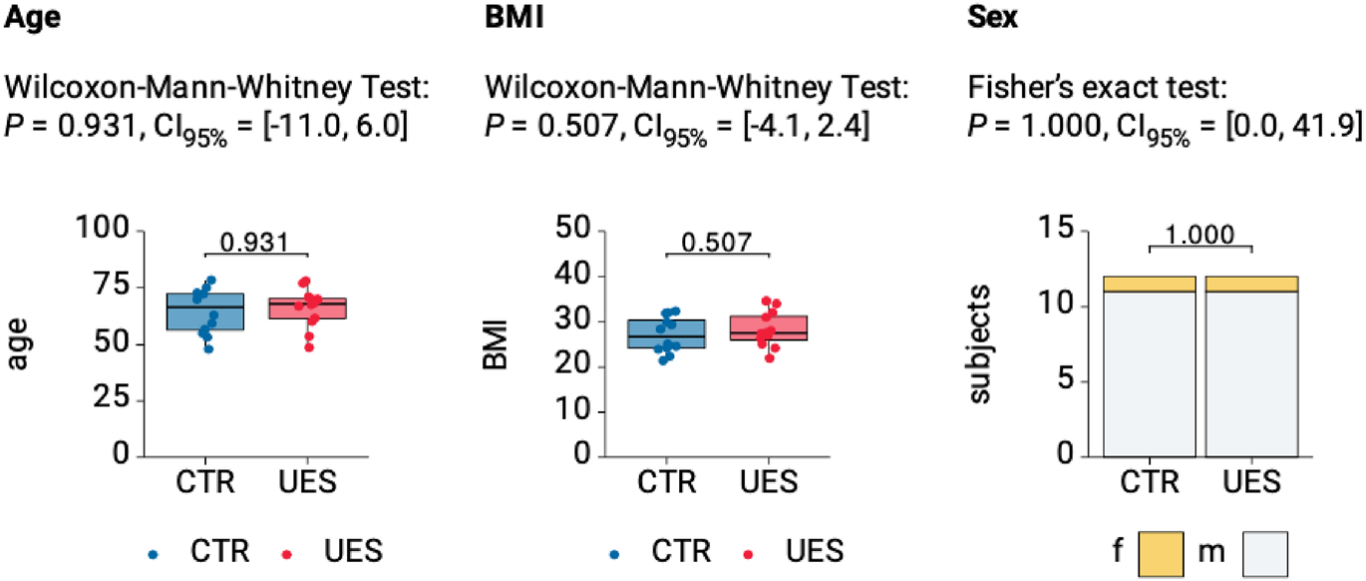
Comparison of demographic characteristics between UES and healthy control group. **A** Age and **B** BMI visualized as boxplots show no significant differ-ences between groups. **C** Sex distribution visualized as stacked bars indicates no significant difference. *P* values and 95% confidence intervals (CI95%) are given for named tests

The UES group consisting of 12 randomly recruited patients (12 eyes) was matched by age, BMI and sex to a healthy control group consisting of 12 patients (12 eyes) (see Fig. 2 and Table 1). Mean patient age was 63.75 ± 10.85 (range 48–78) years in the UES and 66.17 ± 8.61 (range 49–79) years in the control group. In both groups 11 men and one woman were included. The BMI measured 28.2 ± 4.2 (range 22–34.6) kg/m^2^ in the UES and 27.2 ± 4 (range 21.4–32.4) kg/m^2^ in the control group. All values did not statistically differ (Fig. 2). In the UES group, four patients suffered from diabetes mellitus type II, six from arterial hypertension, two from a herniated disc in the past, one from sleep apnea and one from depression. In the control group four persons suffered from diabetes mellitus type II, five persons from arterial hypertension, one person suffered from asthma, one from arthrosis, one from COPD and one from gout. The prevalence of diabetes mellitus type II and arterial hypertension did not statistically differ between the two groups (same number of patients suffering from diabetes mellitus type II, *p* = 0.414 for arterial hypertension).

**Table 1:** Overview of the patient data: age, gender, BMI, best corrected visual acuity (BCVA), treatment history, LCN2 and PTX3 serum values

Patient No	Age (y)	Gender	BMI (kg/m^2^)	BCVA(logMAR)	Treatment history	PTX3 (ng/ml)	LCN2 (ng/ml)
1	49	M	34.6	1	1 × oral steroids for 7 days (total 700 mg) 1 × intravitreal steroids (ozurdex®)	1	74.3
2	78	M	32.1	1	1 × oral steroids for 7 days (total 700 mg) 1 × peribulbar steroids (urbason® 32 mg)	1.4	54.4
3	60	M	22	0.5	1 × oral steroids for 7 days (total 560 mg) 6 × intravitreal steroids (1 × iluvien®, 5 × ozurdex®)	0.1	51.8
4	54	M	27.7	0.3	None	0.5	50.5
5	67	M	24.2	0.6	None	0.7	43.0
6	77	M	34	1.6	1 × oral steroids for 9 days (total 600 mg) 1 × peribulbar steroids (triamcinolon® 40 mg) 1 × intravitreal steroids (ozurdex®)	0.5	71.9
7	68	M	25.1	0.6	1 × peribulbar steroids (triamcinolon® 40 mg)	1.3	51.1
8	71	F	28	0	1 × peribulbar steroids (triamcinolon® 40 mg)	0.6	53.5
9	70	M	27.5	0.6	1 × oral steroids for three days (total 3000 mg)	2.1	165.4
10	68	M	26.3	1.5	8 × intravitreal steroids (1 × iluvien®, 7 × ozurdex®)	0.4	62.8
11	62	M	27	0.1	None	0.8	33.7
12	70	M	31	0.4	1 × peribulbar steroids (triamcinolon® 40 mg)	0.6	75.6

### Blood sample results

**Fig. 3:**
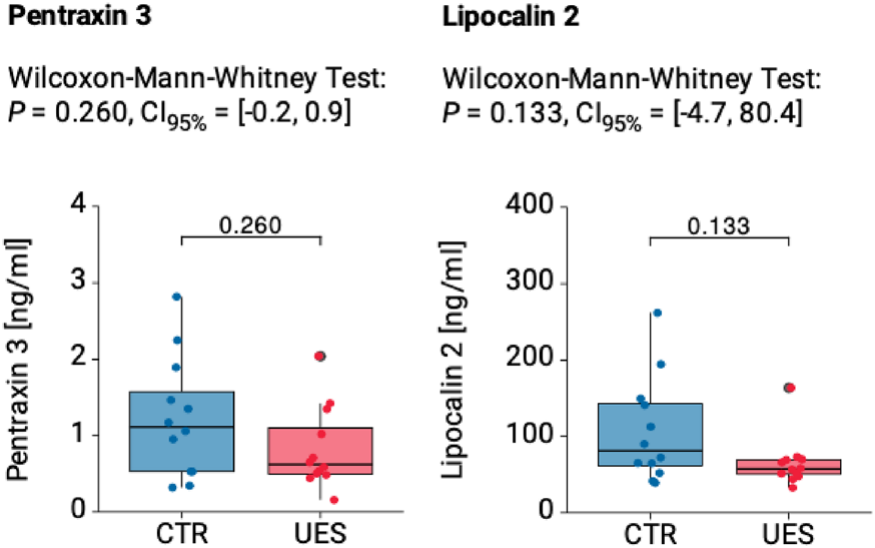
Boxplots showing Pentraxin 3 and Lipocalin 2 blood plasma values in the UES compared to the healthy control group. Both PTX3 (**A**) and LCN2 (**B**) values were markedly lower in the UES group. *P* values and 95% confidence intervals (CI95%) are given for Wilcoxon-Mann–Whitney tests

There was a decrease in PTX3 and LCN2 levels in the UES group compared to the control group (Fig. 3). The UES patient’s mean serum level of PTX3 measured 0.82 ± 0.52 ng/ml (range 0.13–2.1 ng/ml) compared to 1.23 ± 0.77 ng/ml (range 0.3–1.86 ng/ml) in the healthy control group (*p* = 0.260, Fig. 3). The UES patients mean serum value of LCN2 measured 65.74 ± 33.81 ng/ml (range 33.7–165.4 ng/ml) compared to 108.8 ± 67.1 ng/ml (range 40.8–261.9 ng/ml) in the healthy control group (*p* = 0.133, Fig. 3).

The CRP was in the normal range in all subjects (0.3 ± 0.2 mg/dl, range < 0.1–0.8 mg/dl), as well as the liver function parameters except for one patient (GOT: 27.42 ± 9.61 U/L, range 18–55 U/L; GPT: 33.08 ± 10.74 U/L, range 25–62 U/L; GGT: 58.73 ± 78.87 U/L, range 17–292) and kidney function parameters except for one patient (creatinine: 0.99 ± 0.22 mg/dl, range 0.8–1 mg/dl; urea: 38.33 ± 10.08 mg/dl, range 24–54 mg/dl; GFR: 79.83 ± 16.08 ml/min, range 50–100 ml/min).

### Correlation with clinical and imaging data

The mean BCVA in logMAR in the UES group measured 0.68 ± 0.51 (range 0–1.6). The mean CRT was 339.27 ± 53.62 µm (range 250–448.8) and CRV measured 0.47 ± 0.1 mm^3^ (range 0.36–0.64). Mean SFCT was 414.9 ± 60.4 µm (range 274–507). The mean SFCT of the normal control group measured 268.5 ± 53.92 µm (range 165–320). SFCT significantly differed between the two groups (*p* = 0.0003).

BCVA, CRT, CRV and SFCT did not correlate with LCN2 (r = 0.159, *p* = 0.622 for BCVA, r = − 0.108, *p* = 0.738 for CRT, r = − 0.204, *p* = 0.940 for CRV, r = − 0.108, *p* = 0.751 for SFCT), and PTX3 (r = − 0.18, *p* = 0.956 for BCVA, r = 0.122, *p* = 0.706 for CRT, r = − 0.065, *p* = 0.841 for CRV, r = 0.084, *p* = 0.795 for SFCT) serum blood values.

## Discussion

Recent investigations of Matet and colleagues [11] and later on Bousquet and colleagues [10] revealed that lowered LCN2 and PTX3 serum levels could be observed in patients with CSCR compared to healthy controls. The present study shows for the first time that these plasma values similarly tend to be lower in patients with uveal effusion syndrome compared to healthy controls, matched to age, BMI and sex. This novel finding further points towards a pathophysiological link between these two pachychoroidal entities.

A considerable body of studies has already suggested a shared pathophysiology based on clinical observations. However, this is the first study to directly compare the two entities and to take systematically assessed markers into account: As already described above, UES and CSCR are based on similar pathologic findings: It is assumed that patients with UES and recently CSCR exhibit a thickened, rigid sclera [2, 3, 17–19]. This leads to an accumulation of fluid in the choroid [4, 5], which may result in decompensation of the retinal pigment epithelium [15] with exudation of fluid in the subretinal space [2, 20, 21].

For both entities specific pachychoroid features (such as thinning of the Sattler layer and thickened Haller veins up until ciliochoroidal effusion) have been reported [1, 9] [15, 22]. In the present cohort, too a significantly thicker choroid compared to the normal control group could be demonstrated. Onoe et al. reported on a patient with bilateral pachychoroid disease with UES features in one eye and CSCR in the partner eye [23]. Both entities are typically found in middle-aged men and are associated with hypermetropia.

This study adds that there is not only a tendency toward morphological similarities between the entities, but also a measurable trend toward a reduction in the systemic blood markers LCN2 and PTX3 further supporting the hypothesis of a pachychoroidal disease continuum.

LCN2, also known as neutrophil gelatinase-associated lipocalin (NGAL), is a novel circulatory adipokine that is significantly upregulated during inflammation or infection [24, 25]. PTX3, also called tumor necrosis factor-alpha stimulated gene 14 (TSG-14) protein, belongs to the pentraxin superfamily. It is an intracellular, acute phase protein which is part of the innate immune system and plays nonredundant roles in the resistance against diverse pathogens during acute and chronic inflammation [26]. PTX3 is currently emerging as a novel player in neurodegenerative disorders of the retina [27].

In the eye, LCN2 and PTX3, as regulators of inflammation, exhibit a protective effect against oxidative stress on RPE cells [13, 14]: In particular, Parmar et al. reported LCN2 induced expression of the antioxidant enzymes HMOX1 and SOD2 in RPE cells of LCN2 knockout mice. This may lead to an inhibition of the cytotoxic effects of H_2_O_2_ and lipopolysaccharide. He concluded that LCN2 may prevent RPE cell death and might be involved in pro-survival responses during cell stress [13].

Linking this previous work to the herein presented data it can be hypothesized that similar to CSCR, a lack of PTX3 and LCN2 may contribute to RPE disruption and choroidal vascular permeability in UES. A reduced protective effect of these serum markers could play a role in the emergence of the typical RPE alterations and rips with consecutive subretinal fluid inflow and extensive serous retinal detachment at the maximum stage.

Recent studies have demonstrated an enhancing effect of the glucocorticoid dexamethasone on LCN2 and PTX3 transcription, indicating that high protein levels correlate with an activation of the glucocorticoid signaling pathway [12, 16]. Bousquet et al. suggested that an impairment of the adrenal gland or cortisol receptor, or a general insufficiency of the hypothalamic–pituitary–adrenal axis causing a reduced endogenous glucocorticoid secretion and following reduced PTX3 and LCN2 values may be present in patients with CSCR [10, 11]. The herein described findings underscore the need to investigate potential dysfunction in the glucocorticoid-mediated response within the pachychoroid disease spectrum, possibly through further HPA axis function testing.

This study did not detect a direct correlation with BCVA, CRT, CRV, and SFCT. One reason may be that the measured levels are not sensitive enough to capture subtle changes in parameters such as choroidal thickness. To determine whether LCN2 and PTX3 can serve as biomarkers for grading UES severity, a dedicated UES grading system should first be developed that incorporates additional clinical parameters, including the presence of choroidal detachment and scleral thickness. Correlation analysis should then be validated in a larger cohort.

One limitation of the study is that although the difference in laboratory values between the groups showed a clear tendency, it was not statistically significant. This is most likely due to the small number of patients, which moreover does not allow for generalization of the data. In the UES group, a significantly lower spread of values was observed, particularly in relation to LCN2 (Fig. 3), than in the control group, indicating a real reduction in serum values. Considering the rarity of UES with only 1.2 cases per 10 million population [28], the number of UES patients included in this work can still be considered comparatively high. It would be beneficial in the future to merge patient data in a multicenter trial to achieve significant results.

Another limitation of the study is that no histological examinations could be carried out to further examine the scleral structure and to assess potential associations of different types of UES based on scleral histology with PTX3 and LCN2. Longitudinal studies are necessary to investigate the levels and fluctuations of the biomarkers in relation to the severity and activity status of UES to understand disease progression.

## Conclusion

All in all, this is the first study to show that in UES similar to CSCR Pentraxin-3 and Lipocalin-2 levels tend to be lower compared to a healthy control group. Even though due to the low numbers of patients, the difference did not reach statistical significance, the herein presented trend may further support the hypothesis of a similar pathophysiology between the different pachychoroidal phenotypes, along with the demographic and structural similarities of the diseases. Common systemic factors besides structural abnormalities such as a thickened sclera and a small axial length may contribute to the pathologic features. As LCN2 and PTX3 are known to be induced by glucocorticoids, a similar pathophysiology involving the decreased protective effect of LCN2 and PTX3 on the RPE cells in CSCR and UES patients and a dysfunction in the glucocorticoid signaling pathway is currently assumed. PTX3 and LCN2 may serve as potential therapeutic targets or at least as systemic markers for the presence of a pachychoroid disease in the future.

## Data Availability

No datasets were generated or analysed during the current study.
